# Progressive Medicine, Vol, II, June, 1901

**Published:** 1901-07

**Authors:** 


### Books and Magazines.

Progressive Medicine, Vol, II, June, 1901. A Quarterly Digest of Advances, Discoveries and Improvements in the Medical and Surgical Sciences. Edited by Hobart Amory Hare, M. D., Professor of Therapeutics and Materia Medica in Jefferson Medical College of Philadelphia. Octavo, handsomely bound in cloth, 460 pages, with 81 engravings and full-page plate. Lea Brothers & Co., Philadelphia and New York. Issued quarterly. Price, $10.00 per year.

Progressive Medicine is already everywhere known to the reading members of the profession. Under the able editorial management of Professor Hare it well merits the great popularity it has continued to enjoy ever since the issue of the first volume. Its contributors are all men of the highest professional attainments, many of them being distinguished writers and teachers. A point well worth considering is that these men do not furnish a mere series of abstracts of articles taken from the weekly and monthly journals, but make of their contributions original articles in which they express their own views as experts. The contributors to Vol. II are: Dr. William B. Coley on Abdominal Surgery; Dr. John G. Clark on Gynecology; Dr. Alfred Stengle on Diseases of the Blood and Ductless Glands, the Hemorrhagic Diseases, and Metabolic Diseases; and Dr. Edward Jackson on Ophthalmology. R.

